# Optimizing Outcomes in Breast Reconstruction: The Role of Hormonal Therapy Management

**DOI:** 10.3390/cancers17040672

**Published:** 2025-02-17

**Authors:** Eduardo Saorín-Gascón, Óscar Nova-Tayant, Ramón A. Moreno-Villalba, Juan de Dios García-Contreras, Clemente José Fernández-Pascual, Asunción M. Mora-Ortíz, Maria del Carmen Servet-Pérez de Lema, Alba Quiles-Hevia, Antonio Piñero-Madrona

**Affiliations:** 1 Breast Unit, Department of Plastic and Reconstructive Surgery, University Hospital Virgen de la Arrixaca, 30120 Murcia, Spain; 2 Breast Unit, Department of Gynecology, University Hospital Virgen de la Arrixaca, 30120 Murcia, Spain; 3 Department of Anesthesiology, University Hospital Virgen de la Arrixaca, 30120 Murcia, Spain; 4 Breast Unit, Department of General Surgery, University Hospital Virgen de la Arrixaca, 30120 Murcia, Spain

**Keywords:** breast cancer, breast reconstruction, hormonal therapy, postsurgical complications

## Abstract

Background: Breast cancer comprises diverse subtypes with prognostic and therapeutic implications. Hormone therapy plays a crucial role in managing tumors expressing hormonal receptors, but its impact on breast reconstruction outcomes remains unclear. This study aims to evaluate the association between perioperative hormone therapy continuation and the incidence of postoperative complications following breast reconstruction (including autologous, prosthetic, and hybrid techniques), with a focus on identifying specific complication types to refine patient management strategies. Methods: A retrospective analysis was conducted on patients who underwent breast reconstruction following mastectomy for hormone receptor-positive breast cancer. Patients were categorized based on the appropriate discontinuation of hormonal therapy. Clinical data, including patient characteristics, treatment regimens, and complication occurrences, were registered and analyzed. Results: Inadequate suspension of hormonal therapy during the perioperative period was significantly associated with a higher rate of complications, particularly in the immediate and early phases. Complications such as skin alterations, flap failure, seroma development, and clinical infection showed significant associations with the continuation of hormone therapy (*p* < 0.05). No significant differences were observed for other complications. Conclusions: Preliminary findings suggest a potential correlation between the continuation of hormone therapy during the perioperative phase and the development of complications following breast reconstruction.

## Simple Summary

Breast cancer, the most prevalent malignancy in women, involves subtypes with different prognostic and therapeutic implications. Hormone therapy is essential for tumors expressing hormonal receptors, but its impact on breast reconstruction outcomes remains unclear. This study aims to investigate the relationship between perioperative hormone therapy and post-reconstruction complications. Identifying specific complications associated with hormone therapy will inform optimal patient management strategies and refine clinical recommendations. By enhancing the understanding of hormone therapy’s effects on breast reconstruction, this research will contribute to improving outcomes and guiding decision-making for healthcare professionals.

## 1. Introduction

Breast cancer, the most prevalent malignant neoplasm in women [1], has seen significant advancements in its understanding through the identification of different immunohistochemical subtypes, which have crucial prognostic and therapeutic implications [2]. Breast cancer is a heterogeneous disease with distinct histopathological subtypes, primarily invasive ductal carcinoma (IDC) and invasive lobular carcinoma (ILC). These subtypes, along with their molecular classifications (e.g., Luminal A, Luminal B, HER2-positive, and triple-negative), influence treatment strategies and prognosis. Of particular relevance are tumors expressing hormonal receptors (60–70%), corresponding to Luminal A and Luminal B subtypes, as adjuvant hormone therapy (HT) becomes mandatory in such cases [3]. Traditionally, two categories of drugs have been employed for this purpose: selective estrogen receptor modulators (SERMs), such as tamoxifen and raloxifene, which are commonly prescribed for premenopausal women, and aromatase inhibitors (AIs), like anastrozole, letrozole, and exemestane, preferred in postmenopausal patients due to their mechanism of action on estrogen synthesis inhibition. The duration of therapy varies between 5 and 10 years [4], depending on the patient and tumor profile.

The treatment of breast cancer presents substantial challenges for women, extending beyond health and survival concerns to impact body image, self-esteem, sexuality, marital status, family life, work, and social engagement. In light of these considerations, breast reconstruction is now considered a fundamental component of the multidisciplinary management of breast cancer patients undergoing mastectomy and should be systematically offered when oncologically feasible. [5]. However, it is noteworthy that hormone therapy has been associated with potential adverse effects that may interfere with breast reconstruction outcomes. Examples of these effects include elevated rates of deep vein thrombosis or pulmonary embolism, delayed healing processes, and increased complications related to prosthetic materials [6,7,8].

To optimize breast reconstructive outcomes, a comprehensive understanding of the intricacies of hormonal therapy is essential. Surgical teams involved in different reconstructive options have made significant progress in recent decades, benefitting from technical advances and the introduction of new materials. Consequently, protocols for managing hormone therapy during the perioperative period have been established. However, despite these advancements, there remains a dearth of robust evidence to support measures such as temporary discontinuation of hormone therapy [9,10], emphasizing the need for broader studies to develop precise recommendations.

Within this context, the primary objective of this study is to investigate whether maintaining hormonal therapy during the perioperative period correlates with the development of complications after breast reconstruction. Furthermore, this study aims to identify the specific types of complications most commonly associated with this therapeutic approach.

## 2. Materials and Methods

A retrospective cohort study was conducted on a series of women who underwent delayed breast reconstruction procedures at our center between the years 2016 and 2021. Inclusion criteria encompassed patients diagnosed with invasive ductal carcinoma (IDC) or invasive lobular carcinoma (ILC) presenting hormone receptor positivity (Luminal A or Luminal B subtypes). Patients with in situ carcinomas, such as DCIS, were excluded from this study because hormone therapy was not indicated for their primary diagnosis, as well as those with triple-negative or HER2-positive tumors to ensure a homogenous cohort. Despite its retrospective nature, this study obtained approval from the Institutional Review Board (IRB) (registration code: 2023-2-18-HCUVA; date approval: 26 November 2022).

The recorded data for each patient encompassed several variables, including age, presence of high blood pressure (HBP), diabetes (DM), dyslipidemia (DLP), smoking habits, menopausal status, history of deep vein thrombosis (DVT), prior abdominal or breast surgeries, neoadjuvant or adjuvant chemotherapy, radiation therapy, and hormonal therapy. Different types of hormonal therapy were utilized. This study registered both tamoxifen and aromatase inhibitors (anastrozole, letrozole, exemestane). To determine the appropriate suspension of hormonal therapy, this study referred to the latest recommendations from a comprehensive systematic review [11], which suggested suspension durations of approximately 28 days for tamoxifen, 8 days for letrozole, 4 days for anastrozole, and 1 day for exemestane. Hormone therapy was reintroduced two weeks after surgery.

The types of breast reconstruction performed were documented and categorized as follows: autologous tissue reconstructions using DIEP flap or Latissimus Dorsi, prosthetic reconstructions involving implants or expanders, or mixed reconstructions combining autologous and prosthetic techniques. The prosthetic reconstructions in this study included silicone-based cohesive gel implants and tissue expanders from multiple manufacturers, following institutional preferences and patient-specific indications.

Only complications requiring re-surgical intervention or additional procedures were registered in the studies. The recorded complications included pulmonary embolism (PE), deep vein thrombosis, infection, seroma, flap loss, skin alterations (dehiscence and necrosis), prosthetic extrusion, and the development of significant capsular contracture (grades II and IV), classified according to Baker’s scale. Postoperative complications were classified into three groups based on their timing: immediate complications occurring within the first three days postoperatively, early complications within the first three weeks, and late complications occurring after the first three weeks.

This study described the clinical, epidemiological, anatomopathological, and surgical characteristics, as well as the morbidity data, based on the nature of the variables. For quantitative variables, the normal distribution was assessed using the Kolmogorov–Smirnov test. Qualitative variables were described using absolute numbers and percentages.

To conduct the statistical analysis, patients were divided into two groups: a ’maintenance’ group, consisting of those who continued hormone therapy without suspension, and a ’withdrawal’ group, comprising patients who appropriately discontinued hormone therapy according to the recommended suspension duration. The development of surgical complications served as the dependent variable in this analysis.

In the univariate analysis, the Student’s *t*-test was employed to examine the association between qualitative and quantitative variables. For non-parametric variables, the Mann–Whitney U test was used, while the Pearson’s chi-squared test was applied to study the association between qualitative variables. In cases where the expected value count was less than 20%, the Fisher’s exact test was used as a replacement for the chi-squared test. Subsequently, multivariate logistic regression models were adjusted for potential confounders. A *p*-value of less than 0.05 was considered statistically significant.

To identify independent risk factors for postoperative complications, a multivariate logistic regression analysis was performed. Selection of variables for the multivariate logistic regression model was based on clinical relevance and statistical significance observed in the univariate analysis (*p* < 0.1). Potential confounders, including BMI, smoking history, hypertension, diabetes, menopause, radiotherapy, and chemotherapy, were adjusted in the final model to ensure robust identification of independent risk factors. The primary independent variable of interest was the appropriate suspension of hormone therapy (HT) during the perioperative period. Adjusted odds ratios (ORs) with their corresponding 95% confidence intervals (CIs) and *p*-values were reported. Statistical significance was set at *p* < 0.05. The statistical analysis was performed using the SPSS software program (IBM SPSS Statistics for Windows, Version 25.0, IBM Corp, Armonk, NY, USA).

## 3. Results

A total of 230 breast reconstructions were performed on 208 patients, as summarized in Table 1, highlighting the main characteristics of the series. The average age of the patients was 49 years, with a range spanning from 24 to 76 years. The mean duration of follow-up was 52 ± 12.3 months, with a minimum follow-up period of 2 years. Among the patient cohort, 24.2% were active smokers at the time of reconstruction. In terms of venous thrombosis history, only 1.7% had previously experienced thrombotic events, such as deep vein thrombosis (DVT) or pulmonary embolism (PE). Regarding the surgical treatment, 28.6% of patients underwent axillary lymphadenectomy.

The distribution of hormonal therapy varied among the cases analyzed. Specifically, tamoxifen was the most frequently employed, accounting for 75.8% of the cases. Letrozole was used in 12.6% of the cases, anastrozole in 8.2%, and exemestane in 3%.

In terms of the reconstructive techniques utilized, the most prevalent method was the deep inferior epigastric perforator (DIEP) flap, employed in 41% of cases. Silicone breast implants alone were used in 38% of cases, while a combination of silicone breast implants and a latissimus dorsi flap was employed in 13% of cases.

When comparing the characteristics of patients who complied with the established suspension regimen for hormonal therapy and those who did not, there are no significant differences between the two groups, except in the case of total complications, as indicated in Table 2.

The results of the comparative study between groups concerning the occurrence of surgical complications demonstrate that, when hormonal therapy was not properly suspended, a significantly higher rate of complications was observed during the immediate and early postoperative periods. However, no significant differences were observed from 3 months onwards, which corresponds to the late postoperative period, as indicated in Table 3.

Table 4 presents the results of the multivariate analysis for risk factors associated with postoperative complications. The appropriate suspension of hormone therapy (HT) was confirmed as an independent protective factor, significantly reducing the risk of complications (OR = 0.10, 95% CI: 0.04–0.24, *p* < 0.001). Axillary lymphadenectomy also showed a significant association with increased risk (OR = 3.10, 95% CI: 1.34–7.14, *p* = 0.008). Radiotherapy showed a trend towards reduced risk but did not reach statistical significance (OR = 0.49, 95% CI: 0.21–1.13, *p* = 0.094).

Table 5 summarizes the results of the univariate and multivariate analyses for each specific complication. In the univariate analysis, inappropriate withdrawal of hormone therapy (HT) was significantly associated with an increased risk of flap failure (*p* = 0.001), seroma (*p* = 0.008), skin alterations (*p* = 0.022), and infection (*p* = 0.001). After adjusting for potential confounders, including BMI, smoking, hypertension, diabetes, menopause, radiotherapy, and chemotherapy, the multivariate analysis confirmed that HT withdrawal remained an independent risk factor for these complications. Other complications, such as capsular contracture and extrusion, among other minor complications, showed no statistically significant association with HT withdrawal in the adjusted analysis.

## 4. Discussion

Our findings indicate that inadequate suspension of perioperative hormonal therapy may be associated with a higher incidence of immediate and early complications following breast reconstruction. This observation underscores the potential importance of adhering to perioperative hormonal therapy protocols to mitigate these risks.

Patients diagnosed with breast cancer often undergo hormonal therapy for a prolonged duration, typically ranging from 5 to 10 years [11,12,13,14,15]. While these therapeutic approaches have proven effective in managing the disease, it is important to consider the potential negative effects they may have on individuals undergoing breast reconstruction procedures after cancer treatment [11].

Estrogen signaling plays a critical role in multiple stages of wound healing, including the production of growth factors, the proliferation of fibroblasts and keratinocytes, collagen synthesis and degradation, and modulation of the coagulation cascade [16]. However, the administration of exogenous hormonal medications, such as tamoxifen and aromatase inhibitors (AIs), can disrupt the homeostatic levels of estrogens. Tamoxifen, a selective estrogen receptor modulator (SERM), competes with estrogen in the breast and exhibits varied effects in different tissues due to the expression of estrogen receptor isoforms (ERα and ERβ), as well as the presence of coactivators and core-pressors [17] AIs, on the other hand, inhibit the conversion of adrenal androgens into estrogens in postmenopausal women, with letrozole being more effective than anastrozole in reducing plasma estrogen levels [18,19].

Apart from their role in wound healing, estrogens also play a crucial role in maintaining the health of estrogen-responsive tissues, such as the skin [20]. Estrogen deficiency in the elderly contributes to skin aging and impaired healing, while estrogens help preserve skin thickness, collagen, elastic fibers, and hydration. Estrogens regulate the cytokine transforming growth factor-beta 1 (TGF-β1), which is involved in cell proliferation, differentiation, and matrix production, influencing skin healing processes [21]. Estrogens also stimulate the proliferation and migration of various components of the skin through their interaction with dermal fibroblast ER [20]. Given these multifaceted effects of hormonal therapy, especially in breast cancer patients undergoing breast reconstruction, it is crucial to carefully consider the potential benefits and risks associated with these treatments, as they can disrupt the delicate balance of estrogen signaling necessary for wound healing.

Currently, there is a lack of published data regarding the optimal duration for pausing treatment with selective estrogen receptor modulators or aromatase inhibitors without compromising their therapeutic benefits. The adverse effects of tamoxifen are influenced by significant serum levels of certain metabolites, which can remain active for a period of 9 to 14 days [18]. Similarly, available AIs exhibit varying half-lives, ranging approximately from 1 to 4 days [19,22].

Likewise, there is no consensus on the optimal timing to resume tamoxifen therapy after breast reconstruction surgery in cases of suspension. Studies have not thoroughly examined the implications of interrupting tamoxifen for short durations or determined the duration of suspension that can be considered safe without posing oncological implications.

The present study demonstrates that an inadequate interruption of hormonal therapy is associated with a higher incidence of total complications during the immediate postoperative period (up to 3 weeks). However, beyond this timeframe, the difference seems to diminish, possibly due to the fact that patients generally resume hormonal treatment after two weeks, equalizing the long-term effects in both groups.

Recent studies have suggested that perioperative exposure to hormonal therapies, such as tamoxifen or AIs, may increase the risk of postoperative complications [11,13,14]. However, several independent studies have not found a significant association in this regard [14,15]. Despite the lack of consensus on this issue, some recent systematic reviews, such as the one conducted by Spera et al. [11], indicate that temporarily suspending hormonal therapy during the perioperative period may help mitigate these complications, which aligns with the findings of this study.

The multivariate analysis confirmed that inadequate withdrawal of hormone therapy (HT) remains an independent risk factor for postoperative complications, even after adjusting for a comprehensive set of demographic and clinical variables. These findings emphasize the robustness of our results and highlight the importance of strict adherence to perioperative HT protocols. Interestingly, axillary lymphadenectomy emerged as a significant independent risk factor for complications. On the other hand, radiotherapy showed a non-significant trend towards a protective effect, which warrants further investigation in larger cohorts.

Capsular contracture, a prevalent complication in breast implant surgery, refers to the excessive rigidity of the periprosthetic capsule, which leads to implant compression, pain, and deformity. While only one published study [13] has explored the potential effects of hormonal therapy on the prosthetic material, it reported significant differences in higher rates of capsular contracture due to a possible increase in periprosthetic inflammation. However, in the present study, no statistically significant differences were observed in capsular contracture rates (Baker grades III and IV) between the two groups. This finding can be attributed to the fact that capsular contracture typically develops over the medium to long term. From the first postoperative month onward, patients in both groups either maintained or had already resumed hormonal therapy following an appropriate postoperative period, thereby leading to similar pathophysiological effects that may influence the development of contracture. Additionally, Billon et al. [13] conducted their comparisons with a control group of patients who were not susceptible to hormonal treatment. Consequently, based on the examined data, the interruption or continuation of hormonal therapy at the time of surgery does not appear to play a significant role in the development of this complication.

As previously mentioned, the utilization of hormonal therapy, including tamoxifen or aromatase inhibitors, can interfere with the production of growth factors and cell proliferation, which are crucial factors in the process of wound healing [8,16,20,21]. In line with the existing scientific literature, this study demonstrates a statistically significant increase in healing complications, such as dehiscence, cutaneous necrosis, and infection rates, among patients receiving hormonal therapy. These findings are consistent with previous research conducted by Huber et al. and Billon et al. [12,13], which also reported a higher incidence of healing complications in patients undergoing hormonal therapy compared to those not receiving such treatment.

Tamoxifen therapy has been consistently linked with an increased vulnerability to DVT and PE [23,24,25,26,27]. Specifically, women who undergo this therapeutic intervention demonstrate a substantial approximately 2.5-fold higher risk during the initial 5 years following breast cancer surgery, with the first 2 years after treatment initiation representing the period of greatest susceptibility [23]. The association between cancer and DVT/PE is believed to be attributed to the prothrombotic responses of the host to cancer, encompassing inflammatory processes, tissue necrosis, and the tumor cells’ clot-promoting mechanisms, such as the production of procoagulant substances and the release of proinflammatory cytokines [28]. By contrast, aromatase inhibitors have shown a more favorable profile with regard to thrombotic risks compared to tamoxifen. Notably, a study conducted by Coombes et al. [29] revealed that patients treated with AIs had a 50% lower risk of experiencing thrombotic events in comparison to those treated with tamoxifen. While the association between tamoxifen use and an increased risk of venous thrombosis and thromboembolism is well-established, evidence regarding the risk of arterial or venous microvascular anastomotic thrombosis is less conclusive [6,30,31]. This study identifies a significant increase in cases of flap loss in the early postoperative days among patients who did not discontinue hormonal therapy prior to surgery. Among the reviewed studies, only the study by Kelley et al. [14] corroborates these findings in patients taking tamoxifen during the reconstruction process. However, other studies, including the most recent systematic review on this topic [10], have not found statistically significant evidence, although they did observe a slightly higher risk for patients on hormonal treatment.

The impact of hormonal therapy on prosthetic material remains poorly studied in the existing literature. With regard to the loss of implanted prosthetic material, whether it is a prosthesis or an expander, this study reveals a slight increase in risk among patients who did not interrupt their treatment, although this difference is not statistically significant. Only the study conducted by Billon et al. [13] provides data on this issue, but it does not demonstrate a significant association with any of the main complications related to the presence of a foreign body, except for capsular contracture, as previously mentioned.

The occurrence of seroma in patients undergoing breast reconstruction can also be influenced by hormonal treatment, given the role of estrogens in the permeability of blood and lymphatic vessels. In this series, only cases managed with percutaneous drainage or surgical revision were included, revealing significantly higher rates of seroma occurrence in the group that did not follow an appropriate interruption protocol. To the best of our knowledge, no published study to date has specifically addressed this effect, and the few that mention the appearance of seroma [12,13] address it within the context of general wound complications, making it challenging to isolate the potential increased risk.

Other complications recorded, such as hematomas or fat necrosis, do not seem to be influenced by interruption or maintenance of hormonal treatment. This lack of effect may be attributed to the limited number of cases in the sample, which restricts the statistical power of the analysis.

Despite the extensive research conducted thus far, the optimal duration for safely retaining hormonal drugs without elevating the risk of cancer recurrence remains unclear. It is important to note that selective estrogen receptor modulators (SERMs) and aromatase inhibitors (AIs) have demonstrated significant reductions in the incidence of hormone receptor-positive breast cancer recurrence. Therefore, close monitoring and effective communication between oncologists and surgeons are crucial for managing potential complications in breast cancer patients undergoing hormonal therapy.

In conclusion, while our study has contributed valuable insights to the field of hormonal therapy’s influence on breast reconstruction, it is important to acknowledge its inherent limitations. The retrospective design of our investigation inherently poses vulnerabilities to bias and confounding variables. Additionally, it is worth noting that a comprehensive multivariate analysis has not been conducted in this study. Furthermore, the limited sample size, compounded by the rarity of certain scenarios under scrutiny, may impact the generalizability of our findings. These constraints, albeit acknowledged, have underscored the necessity for further comprehensive studies with larger cohorts and prospective designs. Despite these limitations, we believe that our study’s unique focus and rigorous analysis provide a foundation for future research endeavors aiming to elucidate the intricate relationships within the context of hormonal therapy and complications in breast reconstruction.

## 5. Conclusions

Hormonal therapy has emerged as a significant factor associated with an elevated risk of experiencing complications during breast reconstruction, particularly in the initial months following the surgical intervention. Failing to interrupt hormonal therapy could contribute an additional morbidity factor to the surgery. Future prospective trials should focus on investigating the selection of the specific hormonal therapy and determining the optimal duration of suspension before its reintroduction. These studies will provide valuable insights into refining clinical practice and improving patient outcomes in breast reconstruction procedures.

## Figures and Tables

**Table 1:** General characteristics of the patients.

Variable	Total (%)
Age	49 (±11.69)
BMI	25 (±5.1)
Presence of HBP	28 (12.1)
Presence of DLP	25 (10.8)
Presence of DM	9 (3.8)
Menopause	65 (28.1)
Smoking	56 (24.2)
History of previous abdominal surgery	49 (20.9)
History of previous DVT	4 (1.7)
Axillary involvement	66 (28.6)
Neoadjuvant chemotherapy	48 (20.8)
Chemotherapy	145 (62.8)
Radiation therapy	114 (49.4)
Tamoxifen therapy	175 (75.8)
Aromatase inhibitor therapy	
Exemestane	7 (3)
Anastrozole	19 (8.2)
Letrozole	29 (12.6)
Type of reconstruction	
DIEP flap	94 (41)
Latissimus dorsi flap	5 (2.2)
Latissimus dorsi + implant	30 (13.1)
Implant	87 (38)
Expander	12 (5.2)
Postsurgical complications	134 (58.2)

**Table 2:** Characteristics according to hormonal therapy withdrawal.

Variable	Maintains HT (*n* = 64)	Withdraws HT (*n* = 165)	*p* Value
Age	49.58 ± 10.53	49.83 ± 9.94	0.177
BMI	26.29 ± 2.94	25.71 ± 3.77	0.149
Presence of HBP	6 (9.4)	18 (10.9)	0.703
Presence of DM	2 (3.2)	7 (4.2)	0.507
Presence of DLP	7 (10.9)	15 (9.3)	0.701
Menopause	17 (26.9)	47 (28.4)	0.713
History of smoking	20 (31.7)	33 (20)	0.082
History of DVT	1 (1.6)	3 (1.8)	0.882
Previous abdominal surgery	10 (15.8)	37 (22.9)	0.229
Axillary involvement	17 (26.9)	48 (29.8)	0.646
Neoadjuvant chemotherapy	9 (14.2)	39 (24.2)	0.097
Chemotherapy	45 (71.4)	96 (59.6)	0.112
Radiotherapy	37 (57.8)	73 (45.3)	0.084
Postsurgical complications	58 (91)	76 (46)	0.001

**Table 3:** Complications by time of occurrence.

	Maintains HT (*n* = 64)	Withdraws HT (*n* = 165)	*p* Value
Immediate complications	24 (37.5%)	12 (7.3%)	0.001
Early complications	27 (42.2%)	39 (23.6%)	0.05
Late complications	6 (9.4%)	26 (15.8%)	0.211

**Table 4:** Multivariate Logistic Regression Analysis for Postoperative Complications.

Variable	Adjusted OR	95% CI (Lower)	95% CI (Upper)	*p* Value
Age	1.02	0.98	1.07	0.323
BMI	1.03	0.96	1.11	0.415
Presence of HBP	0.89	0.31	2.54	0.828
Presence of DM	0.67	0.12	3.76	0.651
Presence of DLP	1.31	0.45	3.77	0.618
Menopause	0.82	0.35	1.92	0.654
History of smoking	1.47	0.71	3.07	0.301
History of DVT	0.81	0.07	8.77	0.862
Previous abdominal surgery	0.71	0.33	1.54	0.862
Axillary involvement	3.1	1.34	7.14	0.008
Neoadjuvant chemotherapy	1.8	0.81	4.02	0.15
Chemotherapy	0.95	0.45	2.0	0.882
Radiotherapy	0.49	0.21	1.13	0.094
Maintain HT	0.1	0.04	0.24	0.01

**Table 5:** Analysis of the development of different types of complications.

	Maintains HT (*n* = 64)	Withdraws HT (*n* = 165)	Univariate *p* Value	Adjusted OR (95%CI)	Multivariate *p* Value
Capsular contracture	11 (17.2%)	14 (8.5%)	0.058	0.4 (0.17–0.98)	0.046
Skin alterations	22 (34.4%)	33 (20%)	0.022	0.49 (0.25–0.95)	0.033
Flap failure	10 (15.6%)	3 (1.8%)	0.001	0.07 (0.02–0.31)	0.01
Extrusion	16 (25%)	25 (15.2%)	0.081	0.55 (0.26–1.15)	0.112
Seroma	13 (20.3%)	13 (7.9%)	0.008	0.36 (0.16–0.83)	0.017
Infection	19 (29.7%)	19 (11.5%)	0.001	0.33 (0.15–0.69)	0.003
Others	7 (10.9%)	15 (9.1%)	0.670	0.81 (0.3–2.23)	0.686

## Data Availability

The data can be shared up on request.
